# Improved High-Quality Draft Genome Sequence of the Eurypsychrophile *Rhodotorula* sp. JG1b, Isolated from Permafrost in the Hyperarid Upper-Elevation McMurdo Dry Valleys, Antarctica

**DOI:** 10.1128/genomeA.00069-16

**Published:** 2016-03-17

**Authors:** Jacqueline Goordial, Isabelle Raymond-Bouchard, Robert Riley, Jennifer Ronholm, Nicole Shapiro, Tanja Woyke, Kurt M. LaButti, Hope Tice, Mojgan Amirebrahimi, Igor V. Grigoriev, Charles Greer, Corien Bakermans, Lyle Whyte

**Affiliations:** a McGill University, Montréal, Québec, Canada; b DOE Joint Genome Institute, Walnut Creek, California, USA; c National Research Council Canada, Montreal, Québec, Canada; d Altoona College, Pennsylvania State University, Altoona, Pennsylvania, USA

## Abstract

Here, we report the draft genome sequence of *Rhodotorula* sp. strain JG1b, a yeast that was isolated from ice-cemented permafrost in the upper-elevation McMurdo Dry Valleys, Antarctica. The sequenced genome size is 19.39 Mb, consisting of 156 scaffolds and containing a total of 5,625 predicted genes. This is the first known cold-adapted *Rhodotorula* sp. sequenced to date.

## GENOME ANNOUNCEMENT

Members of the genus *Rhodotorula* are pink-pigmented, unicellular yeasts and have been found in diverse environments, including soil, plants, wastewater, spoiled food, air samples, deep-sea hydrothermal vents and glaciers. This genus comprises many extremophiles tolerant to conditions such as high salt, high pressure, anaerobiosis, high concentrations of metals, a pH as low as 1.5, and low temperature (1–4). *Rhodotorula* JG1b is a eurypsychrophilic yeast, with a growth temperature range from 30°C to −10°C and a salinity tolerance of up to 15% NaCl and 12% perchlorate, a powerful freezing-point depressant*. Rhodotorula* sp. JG1b was isolated from University Valley (1,800 m.a.s.l.), located in the upper-elevation McMurdo Dry Valleys, Antarctica (5). This yeast was isolated from ~150,000-year-old ice-cemented permafrost soils (6), which experience permanent darkness, hyperoligotrophy (0.013% total carbon), low water activity (<1% gravimetric soil moisture content), and constant cold temperature (mean annual soil temperature −24°C) (5). Other psychrophilic and psychrotolerant *Rhodotorula* strains have been isolated to date, including *R. aurantiaca*, *R. psychrophila*, *R. psychrophenolica*, and *R. glacialis*, the latter two of which can degrade high concentrations of phenol as a sole carbon source (3, 4).

The objective of this sequencing project was to identify the genes and molecular traits which enable cryophilic organisms, like JG-1b, to thrive in cold and extreme environments. The availability of other *Rhodotorula* genome sequences, including *Rhodotorula graminis* WP1, along with the sequenced genomes of other cold-adapted organisms, will enable researchers to identify novel cold adaptive traits at the molecular level. *Rhodotorula* sp. JG1b is also of interest given the degradative capacity of yeasts and the useful applications for cryophilic yeasts in health, agriculture, and industry.

Using the Covaris LE220 system, 100 ng of DNA was sheared to 270 bp and size-selected using solid-phase reversible immobilization beads (Beckman Coulter). The fragments were treated with end-repair, A-tailing, and ligation of Illumina-compatible adapters (IDT, Inc.) using the KAPA-Illumina library creation kit. The library was quantified using KAPA Biosystem’s next-generation sequencing library qPCR kit. The quantified library was then multiplexed into a pool of 15 libraries and prepared for sequencing utilizing a TruSeq version 3 paired-end cluster kit. Sequencing was performed on the Illumina HiSeq2000 sequencer using a TruSeq SBS version 3 sequencing kit, following a 2 × 150-bp indexed run recipe. Each FASTQ file was quality-control filtered for artifact/process contamination and subsequently assembled together with Velvet (7). The resulting assembly was used to create a long mate-pair library with insert size of 3,000 ± 300 bp, which was then assembled together with the original Illumina library with AllPathsLG release version R47710 (8), to produce a 133.9× coverage main assembly with 156 scaffolds and 171 contigs. The mitochondrial genome was assembled separately with Velvet version 1/2/07 and resulted in 1 scaffold and 1 contig 36,321 bp in length. The assembled genome was annotated using the DOE Joint Genome Institute (JGI) annotation pipeline to predict and annotate 5,625 genes, which are available from the JGI fungal genome portal MycoCosmv (9).

### Nucleotide sequence accession number.

This whole-genome shotgun project has been deposited at DDBJ/EMBL/GenBank under the accession number LQXB00000000.
